# The Efficacy of Intranasal Desmopressin as an Adjuvant in the Acute Renal Colic Pain Management

**DOI:** 10.1155/2014/320327

**Published:** 2014-12-08

**Authors:** Kambiz Masoumi, Ali Asgari Darian, Arash Forouzan, Hassan Barzegari, Fakher Rahim, Maryam Feli, Mehdi Fallah Bagher Sheidaii, Samaneh Porozan

**Affiliations:** ^1^Department of Emergency Medicine, Imam Khomeini General Hospital, Ahvaz Jundishapur University of Medical Sciences, Azadegan Avenue, Ahvaz, Khuzestan Province 6193673166, Iran; ^2^Toxicology Research Center, Ahvaz Jundishapur University of Medical Sciences, Ahvaz 6193673166, Iran

## Abstract

The aim of this study was to compare analgesic effect of intramuscular (IM) sodium diclofenac and intranasal desmopressin combination with IM sodium diclofenac alone in patients with acute renal colic. In this randomized double-blind clinical trial, all patients aged 18 to 55 years who were diagnosed as acute renal colic and met the inclusion and exclusion criteria were randomized into two groups to receive 40 *μ*g intranasal desmopressin spray and 75 mg IM sodium diclofenac combination (Group A) or 75 mg IM sodium diclofenac alone (Group B). The pain score of patients was assessed using a visual analogue scale (VAS) at baseline, 15, 30, 45, and 60 minutes after administration. Of all 159 patients who were assessed for eligibility finally, the results of 120 patients were analyzed. There was no significant difference regarding age and gender between two groups. The baseline VAS score was not significantly different between two groups (*P* = 0.44). The Mean ± SD scores of two groups reduced 15 minutes after drug administration, but this decrease was significantly more in Group A compared with Group B (*P* = 0.02). This pattern continued in minutes 30, 45, and 60 of drug administration. Our results showed that desmopressin could be used as an effective adjuvant in acute renal colic pain management.

## 1. Introduction

Pain is the most common presenting complaint in patients who present to the Emergency Department, which presents in 50–75% of all the patients [1]. Renal colic pain is probably the worst excruciatingly painful event a person can endure. Annually, approximately 1.2 million people are suffering from renal colic, which accounts for about 1% of all hospital admissions [2]. The pain generated by renal colic is primarily caused by the dilation, stretching, and spasm caused by the acute ureteral obstruction. The pattern of the pain depends on the individual's pain threshold and perception and on the speed and degree of the changes in hydrostatic pressure within the proximal ureter and renal pelvis which was related to position of the stone within urinary tract. Severity of pain is related to the degree and site of obstruction, presence of ureteral spasm, and presence of any associated infection, not to the size of the stones. Injectable narcotic analgesics are choice of the medical therapy in patients with acute renal colic. Although opioids are cheap and effective in pain management, they are addictive and cause different side effects, like nausea, vomiting, drowsiness, and impaired consciousness [2]. In addition, nonsteroidal anti-inflammatory drugs (NSAIDs) were effective in treating the pain of renal colic for ages. The most frequent drug, which is used, is injectable sodium diclofenac. Its peak plasma concentration is 10–22 minutes after administration [3].

The need for finding the best drug for renal colic pain management is still crucial. It has been suggested that decrease in diuresis by antidiuretic hormone helps to relieve the pain of renal colic immediately [4–6]. Desmopressin (1-desamino-8-d-arginine vasopressin) is a synthetic structural analog of the antidiuretic hormone, which has stronger antidiuretic effects, and is longer lasting and less vasopressor [7–9]. The mechanism by which desmopressin relieves renal colic pain has not been fully determined but probably is manifold [7]. Desmopressin has been used for renal colic pain management previously, but its effectiveness as an adjuvant to opioids or NSAIDs should be evaluated more.

This study was conducted to compare the analgesic effects of intramuscular sodium diclofenac and intranasal desmopressin combination with intramuscular sodium diclofenac alone in patients with acute renal colic.

## 2. Materials and Methods

This randomized double-blind clinical trial was carried out in the Emergency Department (ED) of Imam Khomeini Hospital, Ahvaz, Iran, from April 2014 to July 2014. All patients complaining of renal colic, aged between 18 and 55 years, presenting to the ED were assessed for the eligibility of the study.

The diagnosis of renal colic was made based on chief compliant (flank pain), history and physical examination, and positive history of renal stone. The presence of renal stone was confirmed in all patients using ultrasound or computed tomography.

Patients with hypertension, coronary artery disease, rhinitis, influenza, coagulopathy, anticoagulant therapy, peptic ulcer disease, asthma, kidney failure, liver failure, pregnancy, use of analgesics within 4 hours and Alpha blockers before admission, history of addiction, surgery on the kidney or ureter, and fluids therapy immediately before admission were excluded. During the study, if a patient could not bear the pain and did not want to continue, he/she was excluded.

Eligible patients were randomized between two groups (A and B) in a 1 : 1 ratio using a computer-generated code. The identities of the study drugs were recorded in a document, folded four times, and then covered for allocation concealment. When a patient was enrolled in the trial, a study nurse retrieved one of the drugs from a box. The medication was prepared by the study nurse and administered by the second nurse who was blinded to the purpose of the intervention. The study drugs were identical in color and appearance; therefore, the patients and study physicians were blinded to identity.

First, using 10-centimeter visual analog scale (VAS), all participants were assessed for severity of pain (10 = the worst possible level of pain and 1 = painless). For this purpose, the emergency physician showed the printed VAS ruler to the patients and asked them to show the number that represents their perception of their current pain. Then in Group A, 40 *μ*g of intranasal desmopressin spray (Minirin, Ferring, Kiel, Germany, 500 *μ*g/vial, 10 *μ*g/puff) equal to 4 puffs of available products (each puff contains 10 micrograms into a nostril alternately) combined with 75 mg intramuscular sodium diclofenac (Osveh Pharma. Co., Tehran, Iran, 75 mg/2cc) was administered. Group B received 75 mg intramuscular sodium diclofenac (Osveh Pharma. Co., Tehran, Iran, 75 mg/2cc) and NACL 0.65% (DECOSALIN 0.65%, Raha Company, Iran, 20 mL nasal spray) intranasal spray (4 puffs; each puff into a nostril alternately).

All participants in both groups were assessed for severity of pain according to VAS standards 15, 30, 45, and 60 minutes after drug administration, and differences in pain level of 2 or more VAS units were considered significant. After 30 minutes, if severity of pain was equal to or more than 5 VAS units, 5 mg morphine sulfate was administered intravenously to the patient as rescue therapy. If any degree of pain persisted after minute 60, an additional dose of morphine sulfate was administered.

Before commencement of the study, we obtained the code of ethics numbered ETH-359 from the Ethical Committee of Ahvaz Jundishapur University of Medical Sciences, and every stage of the study was in accordance with the Helsinki Declaration 1975. Written consent was obtained from all participating patients, and confidentiality of patient's personal details was maintained. This study was registered at Iran's Clinical Trials Registration Centre, and the relevant code was obtained.

*Statistical Analysis.* To consider *α* = 0.05, *β* = 0.2, power = 80%, and the final differences between the two groups at least 2 scores on VAS, the sample size was calculated at least 40 in each group. Continuous variables were summarized as Mean ± SD and categorical variables as ratios. Two-tailed independent *t*-test was carried out to compare quantitative variables with normal distribution and chi-square was done for comparing qualitative ones.

## 3. Results

Initially 159 potential study candidates were assessed for eligibility; however, 36 subjects did not meet the inclusion and exclusion criteria. Three participants refused to finish the study. Eventually, 120 (60 in each group) patients were allocated randomly between the two groups and data from these participants were analyzed.

Baseline characteristics of two groups participants are presented in Table 1.

There was no significant difference regarding baseline VAS pain score (Mean ± SD) between two groups (9.28 ± 0.47 versus 9.35 ± 0.35, *P* value = 0.44).

**Figure 1:**
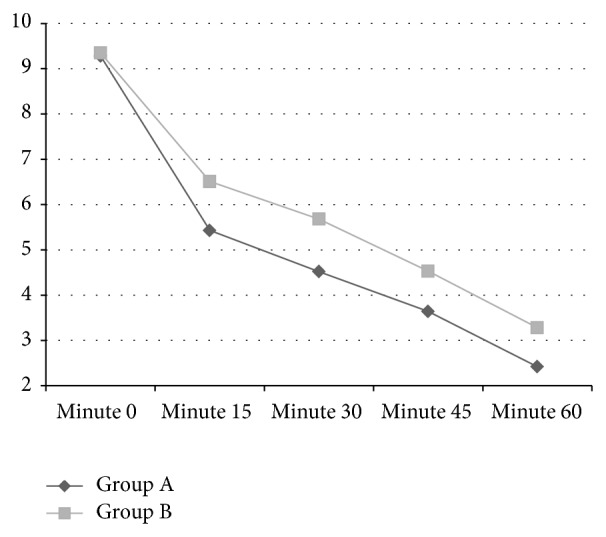
Comparing the pattern of pain score decrease across the time between Group A and Group B patients.

The Mean ± SD scores of two groups reduced 15 minutes after drug administration, but this decrease was significantly more in Group A compared with Group B (*P* = 0.02). This pattern continued in minute 30, minute 45, and minute 60 of drug administration (Table 2, Figure 1).

However, the physician was supposed to administer 5 mg morphine sulfate in 23 and 27 patients in Group A and Group B, respectively, in minute 30, because their pain score was higher than 5. Also, finally 11 patients in Group A and 19 patients in Group B received 5 mg morphine sulfate regarding any degree of pain at minute 60. No side effects were detected in either group.

Findings of ultrasound or CT scanning in two groups showed one to three stones measured 3 mm to 11 mm in urinary tract system from calyces to bladder in which at least one stone has been localized between ureteropelvic and ureterovesical junction and exceptionally two patients with only one calyceal stone in Group A.

## 4. Discussion

Desmopressin is the first line of replacement therapy in the nocturnal enuresis and central diabetes insipidus [7–9]. Likely the antidiuretic mechanism of desmopressin is responsible for making it effective for the treatment of renal colic [7, 10]. Desmopressin suppresses spontaneous contractions of circular smooth muscle fibers in the renal pelvis of rabbits [11]. Some researchers have reported the role of desmopressin in stimulating the release of beta-endorphins by the hypothalamus, which could prove its effective central analgesic [12]. Central mechanism for inducing analgesia can also be an acceptable hypothesis. However, effects of desmopressin on final passage of stones are unknown. Intranasal form of the drug (500 *μ*g/5 mL and 10 *μ*g/puff) is available, which is administered into a nostril. The biphasic half-lives of intranasal desmopressin were 7.8 and 75.5 minutes for the fast and slow phases, respectively. Usually only a single dose of 40 *μ*g/4 puffs of intranasal desmopressin may be prescribed [3].

Our results showed that the combination of nasal desmopressin and IM diclofenac is more effective than IM diclofenac alone in acute renal colic pain reduction. This difference started 15 minutes after drug administration and was maintained during 60 minutes of follow-up.

The pain generated by renal colic is primarily caused by the dilation, stretching, and spasm caused by the acute ureteral obstruction. In the ureter, an increase in proximal peristalsis through activation of intrinsic ureteral pacemakers may contribute to the perception of pain. Muscle spasm, increased proximal peristalsis, local inflammation, irritation, and edema at the site of obstruction may contribute to the development of pain through chemoreceptor activation and stretching of submucosal free nerve endings. Mentioned mechanisms (antidiuretic effects, suppression of spontaneous contractions of circular smooth muscle fibers in the urinary tract system, and stimulating the release of beta-endorphins by the hypothalamus) were considered reasonable causes for effective role of desmopressin in our study.

In 1994, El-Sherif et al. [4] investigated the effectiveness of desmopressin in 18 patients with acute renal colic pain management in 30 minutes. Eight patients had complete pain relief 30 minutes after intranasal desmopressin spray administration. Nine patients need IM diclofenac. Their results are in line with ours, although the small sample size, short follow-up duration, and lack of control group make the judgment difficult.

Constantinides et al., 1998, evaluated desmopressin efficacy in 108 renal colic patients. Over fifty-three percent of their patients respond after 30 minutes. Others received prostaglandin synthesis inhibitors or pethidine for pain relief. They concluded that desmopressin could be used for renal colic pain management [13].

Lopes et al., 2001, compared the efficacy of nasal desmopressin spray with IM diclofenac in three different groups (first, second, and third group received desmopressin, diclofenac, and desmopressin-diclofenac combination, resp.). Their results revealed that pain score reduction was equal in all groups 10 and 20 minutes after drug administration, but at minute 30 there was a slight increase in pain score in the first group. They did not follow patients after minute 30, but in our study pain reduction decreased significantly in desmopressin-diclofenac combination group since 15 minutes after administration and during one-hour follow-up this difference was maintained [14].

In 2010, Kheirollahi et al. [15], in a clinical trial, studied 114 patients who randomly were allocated in two groups including first group which received 20 mg intramuscular hyoscine N-butyl bromide at admission time and second group which received 20 *μ*g of intranasal desmopressin in combination with 20 mg intramuscular hyoscine N-butyl bromide. In the first group, the mean of pain level showed a decrease after 30 minutes but further decreasing did not occur; however, in the second group, the pain consistently decreased and the mean after 60 minutes was significantly decreased. In another study, Roshani et al. [3], in a double-blind controlled clinical trial, determined the effect of the combination of intranasal desmopressin spray and diclofenac sodium suppository in 50 patients with acute renal colic and compared it with diclofenac sodium suppository alone. Patients in the first group received intranasal desmopressin, 40 *μ*g, plus diclofenac sodium suppository 100 mg, and patients in the second group received diclofenac sodium suppository 100 mg plus a placebo spray consisting of normal saline 0.9%. They showed that intranasal desmopressin plus diclofenac sodium suppository caused prompt pain relief with significant decreases in pain scores after 15 and 30 minutes and suggested that intranasal desmopressin spray is a useful supplemental therapy for renal colic in combination with NSAIDs, especially to reduce the use of opioids. In 2012, Moosavi Beladi et al. [2] evaluated 70 patients with acute renal colic that their results showed that desmopressin nasal spray is effective in rapid pain relief for acute renal colic significantly; meanwhile synergistic effect of desmopressin and diclofenac together can cause much better pain decreasing and less using of morphine.

On the other hand, Kumar et al. [16] reported that intranasal desmopressin is not an effective analgesic in renal colic and does not potentiate the effect of diclofenac. However, slow phase of desmopressin action should be considered.

Although several studies have evaluated the desmopressin efficacy in renal colic pain management, there is a disagreement in their results.

Desmopressin has several advantages, such as ease of administration, simplicity of delivery, apparent lack of side effects, and rapid action, which makes it suitable for ambulatory use. It seems that desmopressin is a promising alternative or adjunct to analgesics in patients with acute renal colic, especially in cases that there is contraindication for narcotics or resistant pain to the standard medical therapy. Besides, its biphasic half-life is a considerable advantage. Not only does it have rapid onset effect, but also these analgesic effects maintain for a long duration.

## 5. Conclusions

Our results showed that desmopressin could be used as an effective adjuvant in acute renal colic pain management.

## Figures and Tables

**Table 1:** Comparing age and gender distribution of patients between two groups (A and B).

Variable	Group A (60)	Group B (60)
Age (Mean ± SD), years	32.9 ± 8.24	36.3 ± 9.89
Age (number (%))		
≤35	27 (45%)	32 (53.4%)
36–45	19 (31.6%)	22 (36.6%)
46–55	14 (23.4%)	6 (10%)
Gender (number (%))		
Female	14 (23.4%)	15 (25%)
Male	46 (76.6%)	45 (75%)

**Table 2:** Comparing VAS pain score (Mean ± SD) between two group participants across the time (15, 30, 45, and 60 minutes) after drug administration.

	Group A	Group B	*P* value
Pain score (15′)	5.43 ± 2.70	6.51 ± 2.46	0.02
Pain score (30′)	4.52 ± 2.61	5.68 ± 2.78	0.02
Pain score (45′)	3.64 ± 2.36	4.53 ± 2.15	0.03
Pain score (60′)	2.42 ± 1.94	3.28 ± 2.43	0.03

## Limitations

In this study we did not evaluate the desmopressin analgesic effect in a separate group. Our study has been designed for about 60 minutes and we did not record more information of patients conditions. Also, we did not evaluate intranasal desmopressin effect regarding its slow phase of half-life (75.5 minutes). These problems associated with individual’s pain threshold and perception were considered limitation of our study.
